# First maintenance therapy for COPD in the UK between 2009 and 2012: a retrospective database analysis

**DOI:** 10.1038/npjpcrm.2016.61

**Published:** 2016-11-03

**Authors:** David Price, Marc Miravitlles, Ian Pavord, Mike Thomas, Jadwiga Wedzicha, John Haughney, Katsiaryna Bichel, Daniel West

**Affiliations:** 1 Centre of Academic Primary Care, University of Aberdeen, Aberdeen, UK; 2 Department of Pneumology, Hospital Universitari Vall d’Hebron, Ciber de Enfermedades Respiratorias (CIBERES), Barcelona, Spain; 3 Department of Respiratory Medicine, Nuffield Department of Clinical Medicine, University of Oxford, Oxford, UK; 4 Department of Primary Care Research, University of Southampton, Southampton, UK; 5 Airway Disease Section, National Heart and Lung Institute, Imperial College, London, UK; 6 Research in Real-Life Ltd, Cambridge, UK

## Abstract

Clinical guidelines recommend long-acting bronchodilators as first maintenance therapy for chronic obstructive pulmonary disease (COPD), with inhaled corticosteroids (ICS) reserved for patients with more severe disease and exacerbations. The aim of this analysis was to examine real-life prescribing of first maintenance therapy for COPD in the UK. Data were extracted from the UK Optimum Patient Care Research Database for patients with a first prescription for COPD maintenance therapy between 2009 and 2012 and a diagnosis of COPD at or before the date of the first prescription for COPD maintenance therapy. Routine clinical data including demographics, disease history and symptoms, comorbidities, therapy, hospitalisation rate and exacerbation rate were collected and used to characterise patients stratified by disease severity and Global Initiative for Chronic Obstructive Lung Disease (GOLD) group (A–D). The analysis population included 2,217 individuals (55.4% male, 45.2% smokers). Long-acting muscarinic antagonists (LAMA) as monotherapy were prescribed as first maintenance therapy for 40.2% of patients. ICS were prescribed as ICS/long-acting beta-agonists combination for 29.1% of patients or as monotherapy for 15.5%. ICS (alone or in combination) were prescribed to >40% of patients in each GOLD group. ICS-containing regimens were prescribed to patients with a history of pneumonia and comorbid conditions for whom the risks of ICS therapy may outweigh the benefits. The clinical reality of prescribing indicates that ICS are often prescribed outside current guideline recommendations for many patients newly diagnosed with COPD in the UK. Encouragingly, LAMAs are increasingly being prescribed as first maintenance therapy for these patients.

## Introduction

Over one million individuals in the UK are currently living with chronic obstructive pulmonary disease (COPD), with an estimated 25,000 deaths each year from the disease.^1^ COPD is a complex condition with both pulmonary and extrapulmonary manifestations necessitating a holistic approach to the evaluation and management of patients. In the UK, patients with COPD are largely managed in the primary care setting.^2^ The National Institute for Health and Care Excellence (NICE) guidelines for the assessment and management of patients with COPD advocate a multidimensional approach in agreement with recommendations from the Global Initiative for Chronic Obstructive Lung Disease (GOLD).^3,4^ Broadly, the goals of treatment are to relieve the symptoms of breathlessness, reduce the frequency and severity of exacerbations, improve health-related quality of life, improve exercise capacity and slow disease progression (through smoking cessation).

UK NICE guidelines recommend bronchodilators as initial therapy comprising short-acting beta-agonists or short-acting muscarinic antagonists. For those patients with stable COPD who remain breathless despite using short-acting bronchodilators, long-acting muscarinic antagonists (LAMAs) or long-acting beta-agonists (LABAs) are recommended as maintenance therapy if the forced expiratory volume over 1 s (FEV_1_) is ⩾50%, and LABA+inhaled corticosteroid (ICS) or LAMA for those with FEV_1_ <50% predicted.^4^ These recommendations are in line with GOLD recommendations, which suggest that inhaled ICS therapy should be reserved as an option for those in GOLD groups C or D.^3^ The role of ICS in the management of patients with COPD is still debated. These agents have not been shown to prevent disease progression or reduce mortality in controlled clinical trials and are generally prescribed with the aim of reducing the frequency or severity of exacerbations.^5^ However, many of the inflammatory processes found in individuals with COPD are of a type not amenable to ICS therapy and it remains unclear which patients can be expected to gain meaningful benefit.^6^ An additional challenge when managing patients with COPD is that comorbidities are common and these can impact prognosis.^3^ Comorbid conditions may also influence treatment decisions, particularly with respect to ICS therapy which has been shown to increase the risk for infective comorbidities and be associated with the presence of non-infective comorbidities.^7^ Given these challenges and unanswered questions, it is perhaps unsurprising that there are data to suggest that clinical practice does not always reflect current clinical guidelines in the UK^8^ and other European countries.^9–12^

We reported a previous evaluation of prescribing patterns in the UK among 24,957 patients with moderate airflow limitation (FEV_1_ ⩾50% to <80%) COPD, which corresponds to GOLD stage II.^13^ In this large cohort of patients, around half were receiving an ICS alone or in combination with a LABA or a LABA and a LAMA, a treatment approach not in line with either NICE recommendations or GOLD guidelines. To extend these observations regarding treatment of all patients with COPD, we evaluated treatment selection (prescribing) for patients commencing first maintenance therapy for COPD in the UK. This approach was undertaken in order to investigate whether treatment selection for first maintenance therapy aligns with current local and international treatment guidelines with respect to patient characteristics, medical history, disease severity and the presence of comorbidities, particularly with respect to ICS use.

## Results

**Figure 1:**
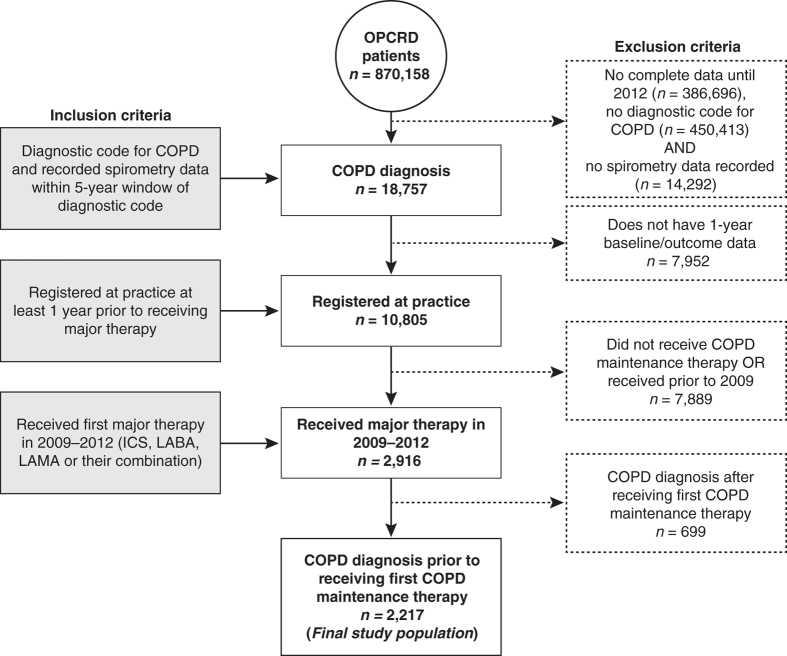
Consort diagram. COPD, chronic obstructive pulmonary disease; ICS, inhaled corticosteroid; LABA, long-acting beta-agonist; LAMA, long-acting muscarinic antagonist; OPCRD, Optimum Patient Care Research Database.

The population for analysis comprised 2,217 individuals with a diagnosis of COPD (confirmed by a post-bronchodilator FEV_1_/forced vital capacity <0.7 for 83% of patients), 55.4% of whom were male, 45.2% were smokers and 46.9% were ex-smokers. Figure 1 illustrates the derivation of the patient cohort for the current analyses. The majority of patients had received their initial diagnosis of COPD and first major therapy for COPD when they were aged ⩾60 to <75 years of age (54.0% and 53.2%, respectively; Table 1).

### First maintenance therapy

The most commonly prescribed first maintenance therapy for COPD was a LAMA (40.2% of patients) followed by an ICS+LABA combination (29.1% of patients). ICS monotherapy was prescribed as first COPD maintenance therapy for 15.5% of patients.

**Figure 2:**
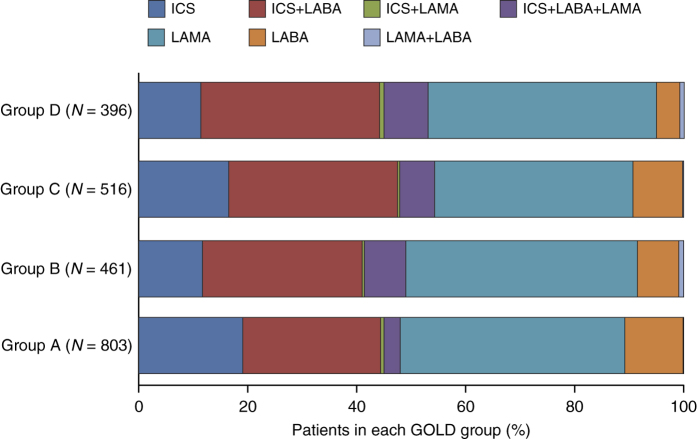
Prescribing pattern for first major therapy class within each GOLD group. GOLD, Global Initiative for Chronic Obstructive Lung Disease; ICS, inhaled corticosteroid; LABA, long-acting beta-agonist; LAMA, long-acting muscarinic antagonist.

Figure 2 details the prescribing pattern for the first major therapy class by GOLD group. Among patients initially assessed as GOLD group A or B, 385/803 (47.9%) and 226/461 (49.0%), respectively, received an initial prescription of an ICS alone or in combination with a bronchodilator. ICS-containing regimens were prescribed to >40.0% of patients in each GOLD group.

### Comorbidities, pneumonia history and prescribing patterns

Among the 2,217 patients newly diagnosed with COPD before or at their first major therapy prescription, 6.8% (*n*=150) had a history of pneumonia, 11.4% had osteoporosis (*n*=252), 15.9% had chronic kidney disease (*n*=353), 17.2% had asthma (*n*=381) and 23.6% (*n*=524) had diabetes (Type 1 or Type 2).

**Figure 3:**
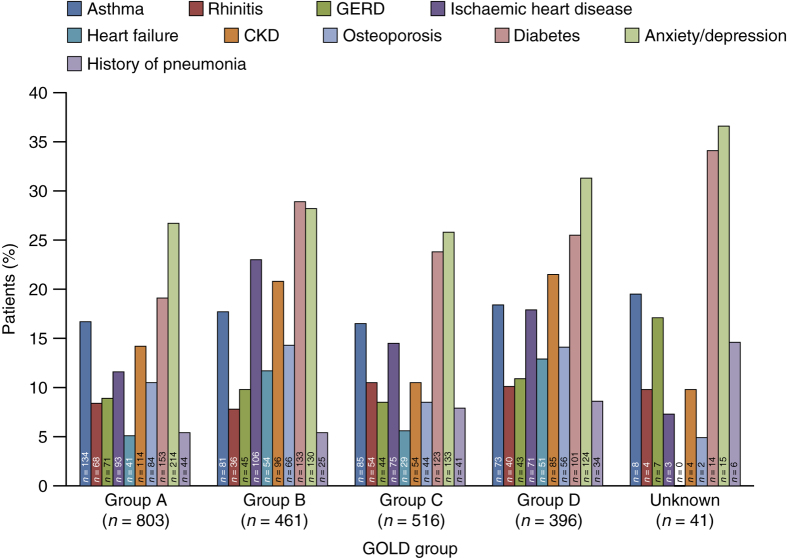
Distribution of comorbidities within each GOLD group. CKD, chronic kidney disease; GERD, gastro-oesophageal reflux disease; GOLD, Global Initiative for Chronic Obstructive Lung Disease.

These comorbidities were apparent among those with GOLD group A and B COPD (osteoporosis 10.5% (84/803) and 14.3% (66/461), chronic kidney disease 14.2% (114/803) and 20.8% (96/461) and diabetes (Type 1 and Type 2) 19.1% (153/803) and 28.9% (133/461), respectively; Figure 3). In all, 5.5% (44/803) and 5.4% (25/461) in GOLD groups A and B, respectively, had a history of pneumonia.

**Figure 4:**
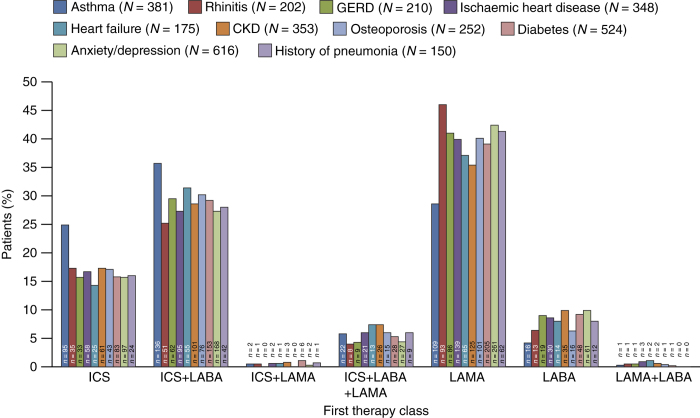
Distribution of comorbidities within each first major therapy class. Proportions are calculated based on the total number of patients reporting each comorbidity and plotted across the first therapy class. CKD, chronic kidney disease; GERD, gastro-oesophageal reflux disease; ICS, inhaled corticosteroid; LABA, long-acting beta-agonist; LAMA, long-acting muscarinic antagonist.

An ICS was prescribed in 255/381 (67%) patients with asthma, either as monotherapy (25%) or in combination with a bronchodilator (42%). Among the 150 patients with a history of pneumonia prior to the first major therapy prescription for COPD, 76 (50.7%) were initially prescribed an ICS-containing regimen (Figure 4). ICS-containing regimens were also prescribed as first maintenance therapy for 134/252 (53.2%) of those with comorbid osteoporosis and 270/524 (51.5%) of those with comorbid diabetes (Type 1 and Type 2).

## Discussion

### Main findings

The data presented here suggest that bronchodilator therapy is increasingly prescribed as first maintenance therapy for patients newly diagnosed with COPD in the UK. However, a relevant proportion of patients continue to receive first maintenance therapy that is not consistent with current recommendations with respect to the prescription of ICS.^3,4^

### Interpretation of findings in relation to previously published work

The results presented here for the UK are consistent with observations in other countries including France, Italy, Norway, Spain, Switzerland and the USA.^9–12,14–17^ Furthermore, baseline demographic data in the current study are comparable to those in similar studies, including much larger real-world populations of patients with COPD.^18,19^ Of particular concern is the observation that 15.5% of patients were prescribed an ICS monotherapy as their first maintenance therapy despite the lack of data, or a licensed indication, to support the use of ICS as monotherapy in COPD at any severity level.^16^

Higher-dose ICS therapy has been shown to increase the risk for side effects, including pneumonia and fractures. However, if the benefits outweigh the associated risks, it may be in a patient’s best interest to receive ICS therapy and this should be decided on a patient-by-patient basis.^20–22^ Importantly, the inappropriate prescribing of ICS exposes patients to the risk of the potentially serious side effects of treatment without the counterbalance of an evidence-based efficacy benefit. Our observations of an overuse of ICS as first maintenance therapy are consistent with a recent cross-sectional evaluation of ICS use among patients with COPD across 41 primary care practices in London.^17^ The authors found that ICS were widely prescribed against current recommendations, including for 38.0% of patients classed as GOLD stage I or II according to the GOLD 2011 guidelines and 33.6% of those classed as GOLD stage III or IV but without exacerbations.^17^ Moreover, >40.0% of those classed as GOLD stage IV, for whom ICS therapy is recommended in current guidelines, were not prescribed these agents. The results of our study confirm and extend the observations reported by White *et al*.^17^ and show that ICS are widely and inappropriately prescribed to patients with COPD from the time of diagnosis regardless of GOLD stage. The reason for the apparent inappropriate prescribing of ICS as first-line therapy may lie, at least in part, in the discrepancy between recommended use of ICS+LABA combinations in clinical guidelines and the actual approved indications for these combinations. GOLD recommend that ICS+LABA be reserved for patients with GOLD group C or D.^3^ However, the licensed indication for these combination products is for patients with severe COPD *with* exacerbations. Consequently, ICS+LABA are approved for use only in a subset of patients classed as GOLD group C or D as the majority of these patients experience only infrequent exacerbations.^23^

### Implications for future research, policy and practice

Our results have shown that ICS prescribing is common even for patients with comorbidities or a medical history of conditions that might suggest against the use of an ICS, including a history of pneumonia, fractures and diabetes (Type 1 and Type 2). The implications of inappropriate ICS prescribing for patients with pre-existing comorbidities has the potential to increase the risk for infective comorbidities such as pneumonia^5,21,24–27^ and non-infective comorbidities including fractures^21,28^ and worsening glycaemic control among those with diabetes.^29,30^ Consequently, patients who meet guideline criteria for ICS prescription (GOLD group C or D with exacerbations) with regard to their COPD should be evaluated carefully for the presence of pre-existing factors such as a history of pneumonia, fracture or Type 2 diabetes. For these patients, the risks of ICS therapy may outweigh the potential benefits. However, in patients with comorbid asthma and COPD, an ICS and bronchodilator combination is likely to be the most appropriate treatment, according to guidelines. A sensitivity analysis excluding those with potential asthma would provide an interesting perspective in a future study.^3^

### Conclusions

In conclusion, there is a need for prescribing to be more in line with current GOLD and NICE guidelines, to ensure the effective and well tolerated treatment of patients with COPD, as the clinical reality of prescribing for patients newly diagnosed with COPD in the UK does not reflect current UK^4^ or international^3^ clinical guidelines. Our data indicate an underuse of bronchodilator therapy and an overuse of ICS therapy in patients in GOLD group A and B. This would suggest that a substantial proportion of patients with COPD may currently be undertreated while also receiving unnecessary ICS therapy with little clinical benefit and increased risk of associated adverse events. Future research should seek to understand the factors that determine the selection of first major therapy for COPD.

## Materials and methods

This was a retrospective, observational database study. Data were extracted from the Optimum Patient Care Research Database in September 2013. This database includes longitudinal anonymous data extracted from 353 primary care practices offering chronic respiratory services in the UK. The database was approved by the Trent Multi Centre Research Ethics Committee for clinical research use (approval reference 10/H0405/3).

### Population for analysis

Data were extracted for patients with a first prescription for COPD maintenance therapy (ICS, LABA, LAMA or their combination) between 2009 and 2012 and a diagnosis of COPD at or before the date of the first prescription for COPD maintenance therapy. Patients were required to have spirometry recorded within 5 years of COPD diagnosis and at least 2 years of continuous data (1 year before and 1 year after the date of the first prescription for COPD maintenance therapy). Patients with a diagnosis of COPD but without sufficient data to determine COPD severity (post-bronchodilator FEV_1_) were excluded from the data set.

### Data collection

The data collection period for the current research was from at least 1 year before, to at least 1 year after, first prescription for therapy, up to a maximum of 4 years. Demographics (including age, body mass index and smoking status), disease characteristics (including severity, therapy before first major COPD therapy, first major COPD therapy, hospitalisation rate, exacerbation rate and questionnaire, and routine modified Medical Research Council score), comorbidities and history of pneumonia were collected.

### Analyses

Patients were stratified according to lung function-based severity (mild, moderate, severe and very severe) and COPD group (A–D; derived from the modified Medical Research Council score), and the demographic and disease characteristics within each stratum were evaluated. First major therapy class was also determined by GOLD group. History of pneumonia and distribution of comorbidities present at or before the initiation of first maintenance therapy were evaluated by GOLD group. The presence of ‘ever’ comorbidities of interest (anxiety/depression, diabetes Type 1 and Type 2, osteoporosis, chronic kidney disease (stage 3 (moderate) to 5 (very severe)), heart failure, ischaemic heart disease, gastro-oesophageal reflux disease, rhinitis and asthma (excluding those with an asthma resolved code)) were categorised by first major therapy class.

### Statistical analysis

Statistical analyses were descriptive throughout.

## Figures and Tables

**Table 1:** Baseline demographics and disease characteristics

*Variable, *N* (%)*	*Total study population (*N*=2,217)*
*Age at initial COPD diagnosis*	
<60 years	497 (22.4)
⩾60 to <75 years	1,197 (54.0)
⩾75 years	523 (23.6)
	
*Age at first major therapy*	
<60 years	403 (18.2)
⩾60 to <75 years	1,179 (53.2)
⩾75 years	635 (28.6)
	
*Time from COPD diagnosis to first prescription for COPD maintenance therapy, median years (range)*	0.4 (0–2.6)
	
*Male gender*	1,229 (55.4)
	
*Smoking status*	
Non-smoker	174 (7.9)
Current smoker	1,002 (45.2)
Ex-smoker	1,040 (46.9)
Unknown	1 (<0.1)
	
*GOLD COPD severity post-bronchodilator*	
1: FEV_1_ ⩾80% predicted	204 (11.1)
2: 50%⩽FEV_1_ <80% predicted	1,038 (56.4)
3: 30%⩽FEV_1_ <50% predicted	499 (27.1)
4: FEV_1_ <30% predicted	99 (5.4)
Unconfirmed COPD (FEV_1_/FVC <0.7)	377 (17.0)
	
*GOLD group*^a^	
A	803 (36.9)
B	461 (21.2)
C	516 (23.7)
D	396 (18.2)
Unknown	41 (1.8)
	
*Moderate and severe exacerbation rate*^b^	
0	1,177 (53.1)
1	598 (27.0)
2	280 (12.6)
⩾3	162 (7.3)
	
*First COPD maintenance prescription*	
ICS	344 (15.5)
ICS+LABA	646 (29.1)
ICS+LAMA	12 (0.5)
ICS+LABA+LAMA	129 (5.8)
LAMA	891 (40.2)
LABA	186 (8.4)
LAMA+LABA	9 (0.4)

COPD, chronic obstructive pulmonary disease; FEV_1_, forced expiratory volume over 1 s; FVC, forced vital capacity; GOLD, Global Initiative for Chronic Obstructive Lung Disease; ICS, inhaled corticosteroid; LABA, long-acting beta-agonist; LAMA, long-acting muscarinic antagonist; MRC, Medical Research Council.

a **GOLD group (A–D):** A is least severe and D is most severe COPD. GOLD group determined according to MRC score. Both routine medical practice recorded and patient questionnaire MRC scores were used with the most recent score taking precedence. The category ‘Unknown’ was assigned to patients with no MRC score available.

b 1 year prior to/at first COPD maintenance therapy prescription, defined as an unscheduled hospital admission/A&E attendance for COPD (definite code) or lower respiratory-related events (i.e., with a lower respiratory read code); OR lower respiratory tract infections treated with antibiotics (definite code); OR acute use of oral steroids (definite plus possible courses); OR antibiotics use with a lower respiratory read code within a ±5-day window.
